# Normal Cortical Myelination in Galectin-4-Deficient Mice

**DOI:** 10.3390/cells11213485

**Published:** 2022-11-03

**Authors:** María Elvira Brocca, Arancha Mora-Rubio, Elena Alonso-Calviño, Elena Fernández-López, Natalia Díez-Revuelta, David Martos-Puñal, Juan Aguilar, Alonso M. Higuero, José Abad-Rodríguez

**Affiliations:** 1 Membrane Biology and Axonal Repair Laboratory, Hospital Nacional de Parapléjicos (SESCAM), Finca La Peraleda s/n, 45071 Toledo, Spain; 2 Experimental Neurophysiology and Neuronal Circuits Laboratory, Hospital Nacional de Parapléjicos (SESCAM), Finca La Peraleda s/n, 45071 Toledo, Spain

**Keywords:** myelin, brain cortex, galectin-4, Lgals4-KO, non-myelinated axon segment, cortical neuron, oligodendrocyte, nerve impulse transmission

## Abstract

Myelin, critical for the correct function of the nervous system, is organized in different patterns that can include long non-myelinated axonal segments. How myelin patterning is regulated remains unexplained. The carbohydrate-binding protein galectin-4 (Gal-4) influences oligodendrocyte differentiation in vitro and is associated with non-myelinable axon segments (NMS) in cultured neurons. In consequence, Gal-4 has been proposed as a myelin patterning regulator, although no in vivo studies have corroborated this hypothesis. We used Gal-4-deficient mice (Lgals4-KO) to study the role of Gal-4 in cortical myelination in vivo. We show that cultured neurons of Lgals4-KO mice form NMS that are regulated as in control neurons. In addition, oligodendrocyte/myelin markers expression measured by biochemical and immunochemical means, and cortical myelin microstructure studied by in-depth image analysis appear unaltered in these animals. Consistently, myelin displays an essentially normal function assessed by in vivo electrophysiology and locomotion analyses. In conclusion, cortical myelin of Lgals4-KO mice does not show any significant defect in composition, organization or function, pointing to a negligible role of Gal-4 in myelination in vivo or, as discussed, to unknown mechanisms that compensate its absence.

## 1. Introduction

Myelin is the lipid-rich membrane that wraps nerve axons, thereby forming a multilamellar and discontinuous insulating sheath. The precise structure of myelin is required for the correct function of the nervous system, maintaining axon homeostasis [1] and supporting fast nerve impulse transmission and plasticity [2,3]. Noteworthy, in addition to the classical myelin organization with myelinated axon portions (internodes) separated by short non-myelinated gaps (nodes of Ranvier), different myelination patterns have been described in brain cortex depending on axon diameter and neuronal type [4], including long non-myelinated axon segments proposed to constitute high-plasticity synaptic platforms [5,6]. 

The majority of molecules involved in myelination are highly glycosylated [7,8], making the regulation and interactions of the glycan moieties fundamental for myelin formation and function [9,10]. In this context, some endogenous carbohydrate-binding proteins (galectins) can selectively bind to specific myelin-related glycan moieties, translating carbohydrate-encoded information into biological signaling relevant for myelin organization and function [11,12,13].

Galectin-4 (Gal-4) is a tandem-repeat type β-galactoside-binding protein, whose structure contains two carbohydrate-recognizing domains connected by a peptide linker [14,15]. Such a structure allows the cross-linking of glycosylated molecules, an important feature for Gal-4 activity [16]. Gal-4 is mainly expressed in the intestinal epithelium where it contributes to stabilize the cholesterol/sphingolipid-enriched membrane domains (membrane rafts) [17], or is secreted to the extracellular mucosa, where it participates as an effector of the innate immunity, as it recognizes and eliminates pathogenic bacteria through its specific interaction with host-like glycosylated antigens [18,19]. Even though Gal-4 is scarcely expressed in the nervous system, in vitro studies have suggested that Gal-4 is involved in the regulation of myelination. For instance, it has been reported that Gal-4 could be released by immature non-myelinated neurons and bound to cell surface receptors expressed by pre-myelinating oligodendrocytes (OLGs), keeping them in a proliferative, de-differentiated stage and allowing axon outgrowth [20]. Later on, the expression of Gal-4 would be down regulated at the onset of myelination allowing the maturation of OLGs. In addition, Gal-4 decorates non-myelinable axon segments (NMS) in neuron/OLGs co-cultures and has been proposed to inhibit the deposition of myelin on the axonal surface [21]. It is important to notice that, even though such an in vitro myelin patterning could be equated to the long non-myelinated axon segments found in brain cortex, so far there are no studies confirming this idea. 

Gal-4 has also been involved in myelin stimulation, either through interaction in the nucleus of OLGs with the glycosylated moiety of Sp1, a transcription factor for myelin basic protein (MBP) [22], or with sulfatide, a Gal-4 high-affinity ligand with a major presence in myelin [23,24]. As a consequence, and according to in vitro data reported so far, it is possible that in different moments of development Gal-4 could act as a negative or as a positive regulator of axonal myelination, although no in vivo studies are available on the actual physiological relevance of Gal-4 in brain myelination.

Here, we present evidence indicating that non-myelinated axon segments, as reported for rat cortical neurons, are similarly produced and regulated in mouse cultured cortical neurons, even in the absence of Gal-4, and that cortical myelin in young Gal-4-deficient mice (Lgals4-KO) does not present any perturbation in composition, organization or function with respect to that of control wild-type animals, suggesting either a minor role of Gal-4 in myelination, or the activation of unknown compensatory mechanism/s that counteract the absence of Gal-4 in vivo and render a normal myelination.

## 2. Materials and Methods

### 2.1. Animals

The murine model used in this research was the Gal-4 knockout mice strain C57BL/6NJ-Lgals4em1(IMPC)J/J (The Jackson Laboratories, Bar Harbor, ME, USA), obtained by CRISPR-Cas9-driven Lgals4 gene deletion (this mice strain will be referred to as Lgals4-KO). Homozygous mice are viable, fertile and do not present anatomical alterations or severe phenotypes that compromise their development or life span (Supplementary Figure S1 for characterization of the Lgals4-KO mice). Wild type C57BL/6NJ mice strain was used as control. Both male and female mice were used for all experiments. All mice were housed and bred in a controlled environment (continuous access to food and water, constant temperature and humidity, and 12 h light:dark cycle) within the animal facilities of the Hospital Nacional de Parapléjicos-Research Center, accredited as “Zoological Core” (Spanish national ref. 5000; regional ref. V-45-168-296) by the Council of Agriculture and Environment of the Junta de Comunidades de Castilla La Mancha. The genotypes of mice were determined by PCR using genomic DNA extracted from the clipped ear tissue. Primers for detection of Gal-4 were as suggested by the provider: WT reverse GGG ACT GTC TCA CCG TCT CA; KO reverse GCA ATC TGA ATC ACA CAG TGG, and common forward TCT CCT ATT GAC CCA TAT CCT TAG. All experimental procedures involving animals were approved by the Animal Bioethics Committee (CEBA) of the Hospital Nacional de Parapléjicos-Research Center.

### 2.2. Neuron Cultures, ICC and Non-Myelinable Segments (NMS) Measurements

Mouse or rat cortical neurons were obtained from E17-18 embryos, respectively, and cultured as previously described [25]. In brief, cell suspensions (150,000 cells) were plated onto poly-L-lysine (PLL, Sigma-Aldrich, St. Louis, MO, USA) glass coverslips in 6 cm Petri dishes (Nunc, Roskilde, Denmark) containing minimal essential medium (MEM, Sigma-Aldrich, St. Louis, MO, USA) supplemented with 10% horse serum (Invitrogen, Carlsbad, CA, USA) (MEM-HS), and maintained in a humidified atmosphere with 5% CO_2_ at 37 °C. After cell attachment (24 h), coverslips were transferred to Petri dishes containing neurobasal medium (Invitrogen, Carlsbad, CA, USA) supplemented with 2% B-27 (Invitrogen, Carlsbad, CA, USA) and 0.5 mM L-glutamine (Sigma-Aldrich, St. Louis, MO, USA). Whenever indicated 7 days in vitro neurons were treated with 1μm Tetrodotoxin (TTX, Alomone Labs, Jerusalem, Israel) for a further 7 days. 

For biochemical purposes, cortical neurons were plated on PLL-covered plastic Petri dishes containing MEM-HS medium, at a density of 2 × 10^6^ cells per dish, and analyzed after different time periods in culture. 

For ICC analysis, cells were fixed with 4% paraformaldehyde for 15 min at RT, aldehyde groups were quenched with 50 mM ammonium chloride for 5 min. Cells were then incubated in blocking solution (2% FBS, 2% BSA, 0.2% gelatin in PBS) for one hour, followed by incubation with primary antibodies against Gal-4, drebrin E, chondroitin sulfate-D/E (CS-D/E), heparan sulfate (HS), voltage-dependent anion channel-1 (VDAC1), poly-sialic acid (PSA), or Caspr (Supplementary Table S3) for one hour at RT or overnight at 4 °C. After extensive washing, cells were incubated with appropriate secondary antibodies for one hour. After labelling under non-permeabilized conditions, cells were fixed again, permeabilized with 0.1% Triton X-100 in PBS for 5 min, washed and blocked as indicated above. Cells were then incubated with anti-β3-tubulin antibodies (Supplementary Table S3) for one hour at RT, washed, and further incubated with appropriate secondary antibodies for one hour. Coverslips were then mounted on glass slides with Mowiol (Calbiochem, Darmstadt, Germany) using DABCO (Sigma-Aldrich, St. Louis, MO, USA) as anti-fade agent. Images were acquired on a confocal microscope (Leica SP5, Leica Microsystems, Germany) or/and a fluorescence microscope (Olympus IX83, Olympus Corporation, Tokyo, Japan).

For NMS length quantification, we used the ImageJ software and the ImageJ plugin Labkit, a random forest-based labeling and segmentation toolkit for big image data [26]. First, NMS in cultured neurons were labeled and segmented with the Labkit plugin. To do so, a subset of randomly chosen NMS was manually labeled by drawing a line over the NMS with the pencil tool. Those labeled pixels were classified as foreground. Image background was also manually labeled with the pencil tool and was classified accordingly. Labkit’s random forest-based pixel classifier was then trained to obtain an entirely segmented image using the default filter settings. The automatically segmented images were then manually curated to correct any segmentation errors, and the classifier was re-trained until an optimal segmentation of the NMS was achieved. This classifier was then used to automatically segment all the images analyzed. The segmented images were used in ImageJ to analyze particles with a minimum size of 2.5 μm^2^ and circularity between 0.0 and 0.6. The generated masks were skeletonized, and the Analyze Skeleton plugin was then used to obtain the length of the NMS. Segments whose length were less than 5 μm were not considered NMS and were therefore not analyzed. The cumulative distributions of the NMS lengths were calculated using GraphPad Prism software (version 9). 

### 2.3. Live Neuron Membrane Crosslinking and Immunoprecipitation (IP)

Cortical neurons from E18 rat embryos plated on 10 cm PLL-covered Petri dishes at high density (15 × 10 ^6^ cells per dish) were processed after 3 days in culture. In brief, live neurons were incubated with anti-Gal-4 antibodies (Supplementary Table S3) at 12 °C for 2 h. Excess unbound antibody was removed by gently washing the cells with Hank’s balanced salt solution (HBSS) and cell surface proteins were cross-linked by incubation with the reversible homobifunctional cross-linker dithiobis(sulfosuccinimidylpropionate) (DTSSP; Thermo Scientific, Rockford, IL, USA), following the manufacturer’s instructions. Reaction was quenched with Tris buffer (pH 7.5), and cells were scraped in cold RIPA lysis buffer (25 mM Tris-HCl pH 7.6, 150 mM NaCl, 1% NP-40, 1% sodium deoxycholate, 0.1% SDS) with a protease inhibitor cocktail (Sigma-Aldrich, St. Louis, MO, USA). Protein extracts were centrifuged for 15 min at 13,000 rpm at 4 °C. Supernatants were incubated with protein-A-Sepharose (GE Healthcare, Uppsala, Sweden) at 4 °C for 1 h, and extensively washed afterwards. The immunoprecipitated complexes were submitted to proteomic analysis.

### 2.4. Tissue Extracts and Raft Purification

Protein extracts for biochemical analyses were prepared at 4 °C by homogenizing in a glass-teflon potter fresh brain cortical tissues from 30-day old Lgals4-KO and WT mice in PBS containing a protease inhibitor cocktail (Sigma-Aldrich, St. Louis, MO, USA). These homogenates were ultrasonicated and centrifuged for 15 min at 13,000 rpm. Supernatants were considered as total extracts. Protein concentration was quantified by the bicinchoninic acid assay (BCA, Thermo Scientific, Rockford, IL, USA). The extracts were further analyzed by WB using antibodies against the oligodendrocyte marker Olig2; the myelin markers MBP, MAG and MOG; and against GAPDH as loading control (Supplementary Table S3). Anti-species HRP-conjugated were used as secondary antibodies and chemiluminiscence measurements were used for quantification (Plus-ECL; Perkin-Elmer Inc., Waltham, MA, USA). 

For membrane fractionation and lipid raft purification, cultured cortical neurons were washed in cold PBS and scraped at 4 °C in 1.5 mL of Hepes buffer (HB; 25 mM Hepes pH 7.4, 2 mM EGTA, 150 mM NaCl, 1 mM DTT) supplemented with a protease inhibitor cocktail (Sigma-Aldrich, St. Louis, MO, USA). Extracts were centrifuged (4 °C, 1500× *g*, 5 min) and the postnuclear extracts were loaded in iodixanol (OptiPrepTM, Sigma-Aldrich, St. Louis, MO, USA) gradients, as described elsewhere [27]. Gradients were centrifuged in a Beckman SW41 rotor (35,000 rpm, 4 °C, 16 h) and 1 mL fractions were obtained. In these conditions, pooled fractions 4 and 5 are lipid rafts. Proteins in rafts were further precipitated with chloroform/methanol and reconstituted for proteomic analysis.

### 2.5. Proteomic Analyses

Protein samples were precipitated and submitted to conventional SDS-PAGE (samples from IP) or isoelectric focusing on IPG stripes followed by SDS-PAGE in Criterion TMTGX TM4-20% Precast Gels (Bio-Rad, Munich, Germany) (membrane raft samples). Gels were stained with Oriole Fluorescent Gel Staining (Bio-Rad, Munich, Germany) and then with Coomassie blue. Protein patterns in gels were digitalized using an Image Scanner III and Typhoon Trio (GE Healthcare, Uppsala, Sweden). Gel bands/spots digestions were performed, as previously described [28]. After digestion, peptides were extracted and resuspended in water/2% formic acid/2% acetonitrile. LC-ESI analyses were performed in a TEMPO nano LC system (AB Sciex, Darmstadt, Germany) combined with a nano LC Autosampler coupled to a modified triple quadrupole (ABSciex 4000 QTRAP LC/MS/MS System). The column used was a C18 Ion Exchange (NTCC-360/75- 3-123). Peptide and protein identifications were performed using the ProteinPilotTM Software V 5.0.1 (Applied Biosystems, Foster City, CA, USA) and the Paragon algorithm. Each MS/MS spectrum was searched against the UniProt_organism_rattus database.

### 2.6. Real Time-PCR

Thirty-day-old mice were sacrificed by cervical dislocation and cortices were immediately dissected on ice. Tissue samples were stored at −20 °C in RNAlater (Invitrogen, Carlsbad, CA, USA) until processing. Total RNAs were extracted using RNeasy Mini Kit (Qiagen, Hilden, Germany) according to the manufacturer’s instructions and adding DNAse (Pure Link DNAse, Invitrogen, Carlsbad, CA, USA) during column washing. The quantity and quality of the RNA were assessed by spectrophotometry (ND-1000 Nanodrop Technologies, Wilmington, DE, USA). cDNA was synthesized with High-Capacity RNA to cDNA kit (Applied Biosystems, Foster City, CA, USA) using 2 µg of total RNA in a 20 µL reaction and following the manufacturer’s instructions.

mRNA expression was measured using RT-PCR with 7900HT Fast Real-Time PCR System (Applied Biosystems, Foster City, CA, USA). Briefly, 10 ng of cDNA were employed in a 20 µL reaction containing TaqMan probes (Applied Biosystems, Supplementary Table S2), TaqMan Master Mix (TaqMan Universal PCR Master Mix No AmpErase UNG, Applied Biosystems) and RNAse free water (Molecular Biology Reagent, Sigma-Aldrich, St. Louis, MO, USA) in 96-well plates (Micro Amp Fast Optical 96 Well, Applied Biosystems, Foster City, CA, USA). After a single step of initial denaturation at 95 °C for 10 min, 40 cycles consisting of 95 °C for 15 s and 60 °C for 1 min were performed. Each sample was analyzed in duplicate. Housekeeping genes (GAPDH and β-actin) were used to normalize mRNA expression levels. The relative amounts of mRNA were calculated using the 2^(−ΔΔCT)^ method [29].

### 2.7. Immunohistochemistry

The 30-day-old mice were deeply anesthetized with sodium pentobarbital and transcardially perfused with 0.9% heparinized saline followed by 4% formaldehyde solution. Brains were carefully removed and post-fixed overnight at 4 °C, incubated in PBS for 24 h, and stored in Olmos cryoprotectant solution (30% ethylene glycol, 0.6 M sucrose and 10 g PVP-40 prepared in 0.1 M phosphate buffer) at −20 °C. Serial coronal sections (40 µm thick) containing the primary somatosensory cortex were collected from bregma −0.94 to −2.18 mm [30], using a vibrating blade microtome (Leica VT1200/VT1200S, Leica Microsystems, Germany).

For 3′,3′-diaminobenzidine staining, brain sections were first rinsed with phosphate-buffered saline (PBS) and incubated in 3% H2O2/50% methanol solution for 15 min to inactivate endogenous peroxidase activity. Non-specific binding of antibody was blocked by incubating sections in a 3.5% BSA and 0.3% Triton X-100 solution. Subsequently, sections were incubated overnight in blocking solution with primary antibody against MBP and proteolipid protein 1 (PLP1) (Supplementary Table S3) at 4 °C and, after extensive washing, with the corresponding secondary antibody (anti-rat or anti-rabbit biotinylated IgG; Supplementary Table S3) for 2 h. The immunoreaction product was visualized after incubation with avidin-biotin complex (Vectastain Elite ABC Kit Peroxidase, Vector Laboratories, Burlingame, CA, USA) for 90 min following by a few seconds with 3′,3′-diaminobenzidine (DAB+ Chromogen, Dako/Agilent Technologies, Santa Clara, CA, USA). Finally, sections were washed with PBS, mounted on slides, dehydrated with ethanol solutions at increasing concentrations, clarified in xylene and coverslipped with DPX mounting medium (Sigma-Aldrich, St. Louis, MO, USA).

For fluorescent staining, sections were blocked with 0.2% Triton X-100, 2% BSA and 8% Donkey Serum for 1 h followed by incubation with the primary antibody against Olig2 (Supplementary Table S3) overnight at 4 °C. After washing with PBS, slices were incubated with fluorophore-conjugated secondary antibody (anti mouse IgG Alexa Fluor-488) for 90 min and nuclei were counterstained with DAPI. Finally, the tissue was coverslipped with Vectashield mounting medium (Vector Laboratories, Burlingame, CA, USA).

### 2.8. Image Analysis, Cortex Segmentation and Cortical Myelin Organization

All images for MBP- and PLP1-labelled brain cortices were mapped with an Olympus BX61 microscope with a 20× objective, using the newCAST software. For Olig2 quantification and microstructural studies of MBP fibers, images were acquired using an Olympus IX83 microscope with 10× and 40× objectives, respectively. Z-stacks images were flattened using an EFI projection (a projection that uses a series of differently focused separate images to calculate a resulting image that is focused on all of its parts) using the Olympus cellSens software.

For all immunohistochemical analyses, regions of interest (ROI) encompassing the somatosensory cortex were manually traced using the polygon-selection tool of the ImageJ software and using the Paxino’s Mouse Brain Atlas [30] as reference. The cortical thickness was then measured at three different positions to account for differences in cortical thickness along the whole S1 area. Perpendicular lines were traced from the upper callosal boundary to the outer layer of the dorsal cerebral cortex, and their lengths were determined using the measure function. The measured value at each position was divided by three and used as landmarks to divide the cortex into three layers (outer, middle and inner) of equal thickness. New ROIs were then drawn for each of the layers (as show in Supplementary Figure S2). This segmentation allowed us to account for differences in myelin distribution between less and more densely myelinated regions [5]. To quantify the MBP and PLP1 immunoreactive area, each ROI was selected, and a background subtraction performed before setting a default threshold that segments the myelin-positive area. The immunoreactive area was expressed as a fraction of the total area assessed. For Olig2-positive cell quantification, a background subtraction and an OTSU threshold was applied to the selected ROI as before, and a particle analysis (size 10–100 μm^2^ and circularity 0.5−1.0) was performed to obtain a count of positive nuclei. Olig2-positive cell numbers were normalized to the total area analyzed.

Myelin microstructure (coherency, fiber length, number of intersections and distribution of orientations) was analyzed using different ImageJ plugins, as described by van Tilborg and collaborators [30] with some modifications. Briefly, two circular (1000 μm^2^) ROIs were positioned within each of the three layers analyzed (Supplementary Figure S2). For each ROI, the background was subtracted, and a default threshold was set to segment all myelin-positive fibers excluding the background. The segmented 8-bit images were converted to masks and a particle analysis (size 1.5 μm^2^ to Infinity) was performed to remove small particles. Finally, the output masks were skeletonized. Using the skeletonized image, several parameters were calculated including the total myelin length using ImageJ’s Measure Skeleton Length tool [31]; and the number of myelinated fiber intersections using the Skeleton Intersection function incorporated in the DiameterJ plugin [32]. To measure the distribution of orientations of the myelinated fibers, the skeletonized image was first horizontally aligned and the Distribution function of the OrientationJ plugin [33] was used to build a weighted orientation histogram for each ROI (parameters: σ = 2, cubic spline gradient, min coherency 10%, and min energy 10%). The histogram values were normalized to the maximum value within the distribution, and average orientation distributions were then calculated. The coherency of myelinated fibers was measured using the Measure function of the OrientationJ plugin directly over the selected ROIs without any prior segmentation. For MBP and PLP1 immunohistochemical analyses, 6 WT and 6 Lgals4-KO animals were used. For Olig2 analysis 4 animals of each strain were used. Both hemispheres per section and 4 sections per animal (collected every 320 μm) were analyzed, and the mean value was calculated for each measured parameter.

### 2.9. Brain Volume Analyses by NMR Imaging

To obtain brain images 4 WT and 5 Lgals4-KO mice (aged 45 and 90 days) were anesthetized by means of a gaseous stream of Isoflurane/Oxygen with continuous monitoring of respiration and body temperature. The study was carried out on a Bruker Biospec 70/30 (7 Tesla) NMR equipment, using a high-resolution mouse cryoprobe in order to increase by 2.5 times the resolution of regular probes operating at room temperature. T2-weighted anatomical images were acquired from Bregma 2.3 mm to Bregma −4.6 mm, with a z-depth of 6.9 mm (Supplementary Figure S1). The volume of the brain was determined between Bregma 2.3 mm and −3.7 mm, comprising a total depth of 6 mm that goes from the prefrontal cortex to the dorsal hippocampus. Whole brain contour segmentation (ROI) was performed using the “Segmentation Editor” ImageJ plugin. For volume calculation, the voxel counter and manager 3D plugins of the same software were used. For cortical thickness measurement, the analysis was restricted from Bregma, 1.78 mm, to Bregma, −2.46 mm, comprising a total depth of 4.24 mm. Cortical outer and inner boundaries were delimited and a line was traced perpendicular to the surface in order to determine the thickness of the cortex.

### 2.10. Electrophysiological Measurements In Vivo

A cohort of 33 animals (aged from 2 to 6 months) was used for electrophysiological experiments, from which 12 were wild type (control group) and 21 were Lgals4-KO (Gal4-KO group). Animals were anesthetized with isofluorane (1.5–2% in O_2_) and placed in a stereotaxic frame (SR-6 Narishige Scientific Instruments, Tokyo, Japan) for cranial surgery. Body temperature was kept constant at 36 °C using a homeothermic blanket (CIBERTEC, SL. Madrid, Spain). Lidocaine (2%) was applied subcutaneously in the cranial area as local anesthesia before removal of the skin and exposure the cranial bone. Small craniotomies were made over stereotaxic coordinates of hippocampal region of pyramidal layer in CA1 (AP −1.5, ML 1.2, DV 1.2–1.5 mm) in the right hemisphere and contralateral hippocampal region of CA3 (AP −1.1, ML 0.8, DV 1.5–1.7 mm) [30]. In addition, the correct locations of both electrodes were determined by optimizing physiologically the CA1 responses to stimulation in CA3. Electrophysiological recordings from CA1 were obtained using a tungsten electrode (TM31C40KT, 4-MΩ impedance at 1 kHz or TM31A50KT, 5-MΩ impedance at 1 kHz: World Precision Instruments Inc., Sarasota, FL, USA) in AC mode using a system composed by a pre-amplifier, an amplifier, and a filter (Neurolog, Digitimer Ltd., Welwyn Garden City, UK). In our case, the signal was filtered to 0.5–3000 Hz. Electrical stimulation in CA3 was applied using a concentric bipolar stainless-steel electrode, with 0.5 mm separation between outer electrode (330 µm diameter) and core electrode (100 µm diameter) (Microprobes for Life, Gaithersburg, MD, USA). Square electrical pulses were applied using a Master-8 electrical stimulator (AMPI. Jerusalem, Israel) and the isolator pulse Iso-Flexunit (AMPI). Stimulation pulses were of 0.05ms duration, with intensities ranging from 0.1 to 1 mA, at 0.5 and 5 Hz frequencies. The stimulation protocols consisted of increasing steps of intensities applied in CA3 to detect the threshold of response in CA1, which was considered the stimulation intensity at which the evoked response in CA1 show a populational response (acute negative wave) over the positive populational synaptic response. Electrophysiological recordings were digitalized at 20 KHz by A/D converter CED Power 1401 Plus using Spike 2 software (Cambridge Electronic Devices. Ltd., Cambridge, UK) and stored in a computer for further analysis. Offline analyses were carried out with the Spike 2 software to obtain averaged populational spike recorded in the pyramidal layer of CA1 in response to CA3 stimulation with unfiltered signal. In a second step, Spike2 software utilities were applied to process the signal (0.5 ms DC Remove) and to isolate action potentials of neuronal population close to the electrode (0.1 ms signal Smooth). For a precise identification and quantification of action potentials, a voltage threshold was applied, which was settled at five times the standard deviation of background noise. Action potentials were stored as events in a new channel for posterior quantification of CA1 responses using peri-stimulus time histogram (PSTH). All responses were obtained from responses to 30–50 pulses at the same stimulation intensity.

### 2.11. Behavioral Tests

For the behavioral tests conducted (open field and rotarod), all mice were naïve to the testing devices and were tested in a random order by an investigator blinded to the animal genotype. One week prior to testing all animals were handled daily for at least 5 min. For acclimatization, mice were placed in the testing room under a dim red light for 1 h, and all tests were performed during the animal’s light cycle. As hormonal state can affect behavioral performance, oestrous cycle was monitored via vaginal cytology and behavioral testing began during the oestrous phase. Female and male animals were tested on separate days.

Spontaneous locomotor activity was evaluated using the open field test. For this test, 47 WT females, 34 KO females, 29 WT males and 32 KO males between 3–7 months of age were used. Briefly, after habituation, the animals were placed in the center of a squared grey arena (50 cm × 50 cm × 50 cm, Noldus, Wageningen, The Nehterlands) and allowed to freely explore it during 10 min. The EthoVision XT detection system (Noldus, Wageningen, The Nehterlands) was used to record and track the moving mice. The variables analyzed were total distance travelled, mean velocity and maximum velocity. The arena was thoroughly cleaned with 70% EtOH in order to minimize olfactory cues between testing sessions.

Accelerating rotarod test was used to assess motor skill learning and motor performance. For this test 12 WT females, 6 Lgals4-KO females, 9 WT males and 5 Lgals4-KO males, 7 months-old mice were used. The equipment was thoroughly cleaned with water and mild detergent between each animal change. In this test, mice are trained to maintain balance on a horizontal rotating rod (Rota-Rod 47600, Ugo Basile, Gemonio, Italy) [34]. For habituation, mice were placed on the static rod for 1 min and then at constant speed (4 rpm) for 1 min during which time the mice were placed immediately back onto the rotarod if they fell. Habituation was carried out only once the first day before the first trial. Then, mice were tested in three trials with 15 min of rest between them, in 4 consecutive days. In each trial the speed was accelerating from 4 to 40 rpm during 4.4 min period, and the total length of the test was a maximum 5 min. The latency to fall was recorded and averaged from the three trials performed on the same day.

### 2.12. Statistical Analyses

Normal distribution was determined using the Shapiro–Wilk test or Kolmogorov–Smirnov test. For normally distributed data with equal variances, a two-tailed unpaired Student’s *t* test was used to compare mean differences between the two groups. A two-way ANOVA (strain × gender) followed by a Tukey post hoc test was used when two factors were analyzed. When normality of the residuals and the homogeneity of variances across groups was not assumed, an unpaired *t*-test or a Mann–Whitney test was conducted.

For time-series experiments, a two-way ANOVA with repeated measures followed by Bonferroni post hoc test was used. Data represent the mean ± SEM. Statistically significant differences are indicated as follows: * *p* < 0.05; ** *p* < 0.01; *** *p* < 0.001. Statistical details for specific experiments, including exact n values and statistical tests used can be found in the figure legends.

## 3. Results

Several in vitro studies have involved Gal-4 in myelin formation and organization, although no in vivo studies have corroborated this so far. We have approached this issue using Lgals4-KO mice as a model to study myelination in a Gal-4-deficient background.

### 3.1. Unaltered Formation and Regulation of Axon NMS in Cultured Gal-4-Deficient Neurons

**Figure 1:**
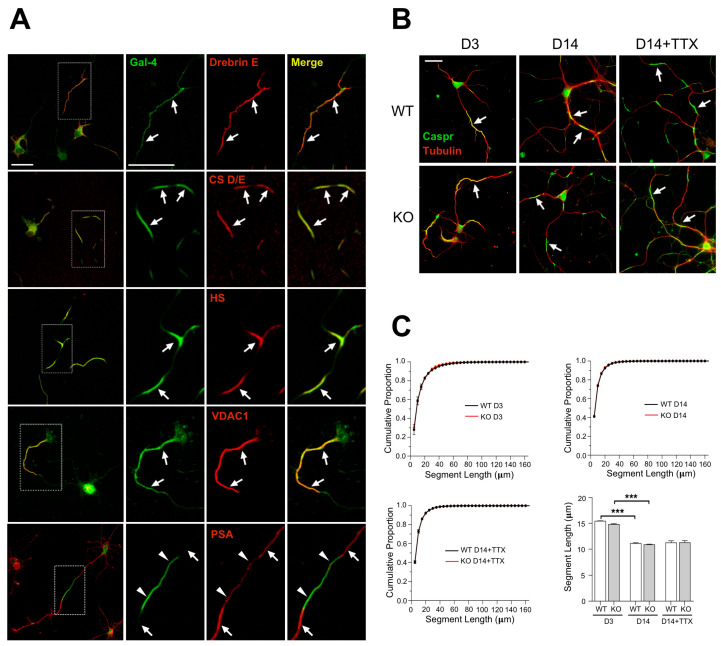
(**A**) Components of the NMS corroborated by IF. Rat cortical neurons in culture, analyzed by double immunofluorescence under non-permeabilizing conditions for the NMS marker Gal-4 and the indicated molecules (Supplementary Table S1). NMS are indicated by arrows. Magnified ROIs are indicated by dotted line boxes in the left column. Caspr and contactin are not shown as they have been associated with NMS in former reports [21]. PSA-NCAM is an example of expression in axonal segments alternative to NMS (arrowheads in bottom panels). (**B**) Cortical neurons from WT (top row) and Lgals4-KO (bottomrow) mice, sequentially immunostained against Caspr (green) before permeabilization and against β3-tubulin (red) after permeabilization. The length of the NMS expressing Caspr was measured at 3 and 14 DIV, or at 14 DIV after 7 days treatment with TTX (D14+TTX). NMS are indicated by arrows. (**C**) Cumulative proportion distributions of the NMS lengths at 3 and 14 DIV (D3 and D14, respectively), or at 14 DIV after 7 days treatment with TTX (D14+TTX). A two-way ANOVA followed by Tukey post hoc test showed no significant differences. Bottom-right bar graph displays NMS length at the same times and treatment in culture. Data are means + SEM (n = 3 independent experiments, 6074 to 45360 NMS were measured; *** *p* > 0.001). (Scale bars are 20 μm).

The occurrence of long axonal segments that are not myelinated in cortical neurons has been described [5,6] in vivo. Non-myelinable axon segments, or NMS, were also revealed by Gal-4 labelling on the surface of non-permeabilized cultured cortical and hippocampal neurons [21,35]. Whether both types of myelin-free axon segments are related is not known. In spite of this, Gal-4 was proposed as a putative organizer of myelination that could locally inhibit myelin deposition at the NMS [21]. In order to evaluate the role of Gal-4 in the formation of NMS, we first investigated which other molecular components are part of the NMS. As these segments cannot be purified by biochemical means, we identified putative molecular candidates using different approaches: (i) Crosslinking and IP of Gal-4-associated proteins followed by proteomic analysis; (ii) co-localization by immunofluorescence of node/paranode components and anionic glycoconjugates with Gal-4 under non-permeabilizing conditions and, (iii) proteomic analysis of Gal-4-containing membrane raft fractions purified from neurons in culture. Some of those candidates were effectively associated with NMS by co-immunostaining in non-permeabilizing conditions with the NMS marker Gal-4 (Figure 1A, Supplementary Table S1). Those showing the clearest signal in immunofluorescence, i.e., Caspr or VDAC, were selected to visualize the formation and to follow the regulation of NMS in a Gal-4-deficient background, comparing cultures of cortical neurons from WT and Lgals4-KO mice.

NMS are regulated along neuronal differentiation in vitro [21], but it was unknown whether they could be equally formed and regulated in the absence of Gal-4. Caspr immunostaining under non-permeabilizing conditions showed that NMS in cultured cortical neurons from Lgals4-KO mice are formed similarly to those in WT mice neurons (Figure 1B, D3 and D14). In addition, the distribution curves of NMS lengths in neurons of both mice strains largely overlap at the different times in culture (3 and 14 div) (Figure 1C, upper graphs), and the variations of their average length with time in culture are also comparable (Figure 1C, bar graph). Neuronal activity also regulates myelination by influencing OLG proliferation, differentiation, and myelin formation; and the concept of activity-driven myelin plasticity related to cognitive status, is acquiring relevance [36,37,38,39]. Since maturing neurons become electrically active, and NMS are regulated in size along neuronal maturation, we explored the possibility that changes in neuronal activity could also regulate NMS size, making neurons more or less “permissive” to myelination as the size of the NMS changes. We also tested if such a regulation could be different in the absence of Gal-4. To check this, the activity of 7 DIV cultured neurons was lowered using the voltage-gated Na+ channel blocker tetrodotoxin (TTX), for further 7 days. NMS average length tends to increase in WT neurons upon TTX treatment (Figure 1B,C, bar graph D14+TTX). Importantly, the absence of Gal-4 in neurons from KO mice does not alter the distribution curves of NMS length (Figure 1C, bottom-left graph), or the trend toward longer NMS lengths observed in WT neurons upon TTX treatment (Figure 1B,C, bar graph).

These results indicate that NMS formation, and their regulation with neuronal differentiation can still occur and be key for myelin organization independently of Gal-4 expression.

### 3.2. Normal OLG Maturation in the Absence of Gal-4 In Vivo

**Figure 2:**
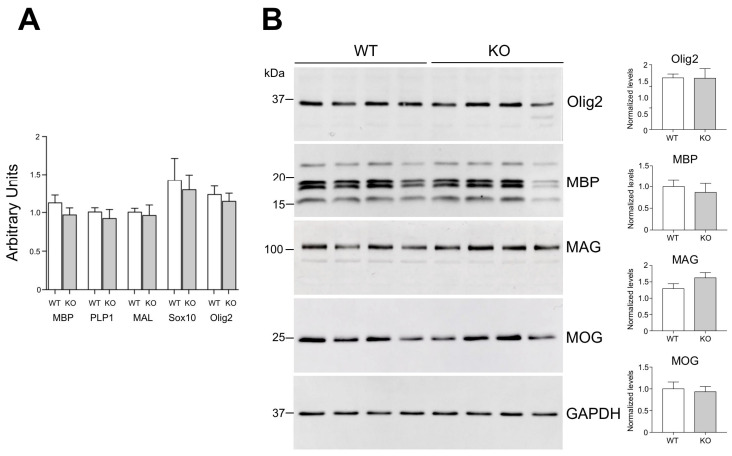
Oligodendrocyte/myelin markers expression in cerebral cortex. (**A**) mRNA expression levels of OLG lineage markers Olig2 and Sox10, and of mature-myelinating OLG marker MBP, PLP1 and MAL measured by RT-PCR. No significant differences for any of the markers were observed when comparing WT to Lgals4-KO (KO) mice according to the Student’s *t*-test. Values are means of normalized mRNA expression levels + SEM (MBP, n = 11; Olig2, n = 12; Sox10, n = 6; PLP1 and MAL, n = 5). (**B**) Protein levels of myelin markers measured by WB. Cortical extracts from 4 WT animals (4 lines on the left) and 4 Lgals4-KO animals (4 lines on the right) were analyzed. GAPDH was used as loading control. In graphs, values are means of normalized densitometric measures + SEM. In agreement with the results in (**A**), no significant differences were observed between WT and Lgals4-KO animals for the protein expression of any of the assayed markers according to the Student’s *t*-test.

Gal-4 has been shown to modulate OLG proliferation/maturation in vitro [20,22,40], thus we checked whether the absence of Gal-4 correlated with altered OLG maturation in vivo. mRNA expression levels of OLG lineage markers Olig2 and Sox10, and of mature-myelinating OLG marker MBP, PLP1 and myelin and lymphocyte protein (MAL) were measured by RT-PCR and compared in cerebral cortices of WT and Lgals4-KO mice. No significant differences were observed for any of the markers (Figure 2A). In order to validate our RT-PCR results, Olig2 and MBP plus the mature OLG markers myelin oligodendrocyte glycoprotein (MOG) and myelin-associated glycoprotein (MAG) were measured by Western blot in cortical extracts from WT and Lgals4-KO animals. Similar expression levels were observed for all the studied molecules when WT and Lgals4-KO mice were compared (Figure 2B). These results corroborated the expression profiles from RT-PCR experiments and, in contrast to previous in vitro evidence, suggest that the absence of Gal-4 is not enough to affect OLG maturation and myelin production in brain somatosensory cortex.

### 3.3. Unchanged OLG and Myelin Distribution in Somatosensory Cortex of Lgals4-KO Mice

**Figure 3:**
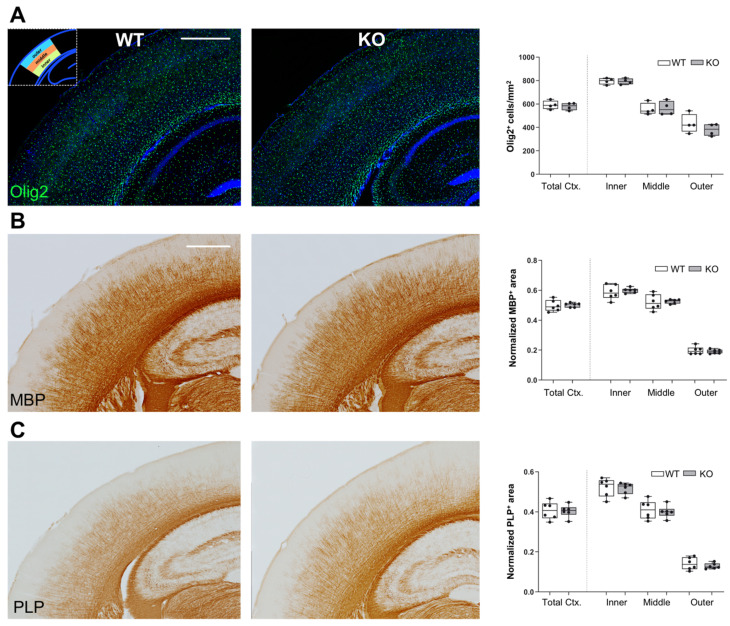
Unchanged distribution of OLG and myelin markers in the cortex of Lgals4-KO mice. All stainings were performed on somatosensory (S1) cerebral cortex of wild-type (WT) and Lgals4-KO (KO) mice. (**A**) Immunofluorescence staining for oligodendrocyte marker Olig2 (green). The graph on the right-hand side displays the density of Olig2-expressing cells (number/area) in the total S1 cortex (Total Ctx.) and in the three segments (inner, middle and outer; see methods and Supplementary Figure S2) depicted in the top-left inset. Data are arranged as a min-to-max box plot (WT n = 4; KO n = 4). (**B**,**C**) Immunohistochemical stainings for myelin markers MBP and PLP1, respectively. The graphs on the right-hand side of these panels display the normalized area stained by each marker following the same segmentation as in (**A**). Data are arranged as a min-to-max box plot (WT n = 6; KO n = 6). Dots in bar graphs represent the mean value of all the sections analyzed per animal. No significant differences were observed in any case between WT and Lgals4-KO mice according to Student’s *t*-test. (Scale bars are 500 μm).

To analyze whether the distribution of OLGs and/or myelinated axons is altered in Gal-4-deficient brain cortex, we used slices of brains from 30 days old WT and Lgals4-KO mice to measure different myelin-related parameters in the whole cortex and in the segmented inner, middle, and outer zones of equal thickness (Supplementary Figure S2). Such a segmentation was made to adapt the quantification parameters to areas showing very different myelin density distribution [5]. First, we evaluated the presence and localization of OLGs by immunofluorescence using anti-Olig2 antibodies. Quantification of Olig-2-positive cell bodies showed no significant differences in total cortex cell density between WT and Lgals4-KO, and also a similar cell density distribution was found when comparing the different segmented zones of WT and Lgals4-KO mice (Figure 3A, graph). These results indicate that the absence of Gal-4 does not affect the quantity or the localization of cortical OLGs. Furthermore, we also compared the level and distribution of mature myelin in somatosensory cortex by immunohistochemical labelling of MBP and PLP1. Positively (threshold) labelled areas normalized to total areas were calculated (Figure 3B,C). No differences were detected in total cortical MBP and PLP1 immunoreactive area when comparing WT and Lgals4-KO mice (Figure 3B,C; Total Ctx in graphs). Similarly, there were no significant variations in MBP and PLP1 immunoreactive area, and therefore distribution, in all three different cortical segments (Figure 3B,C; inner, middle, and outer in graphs). 

**Figure 4:**
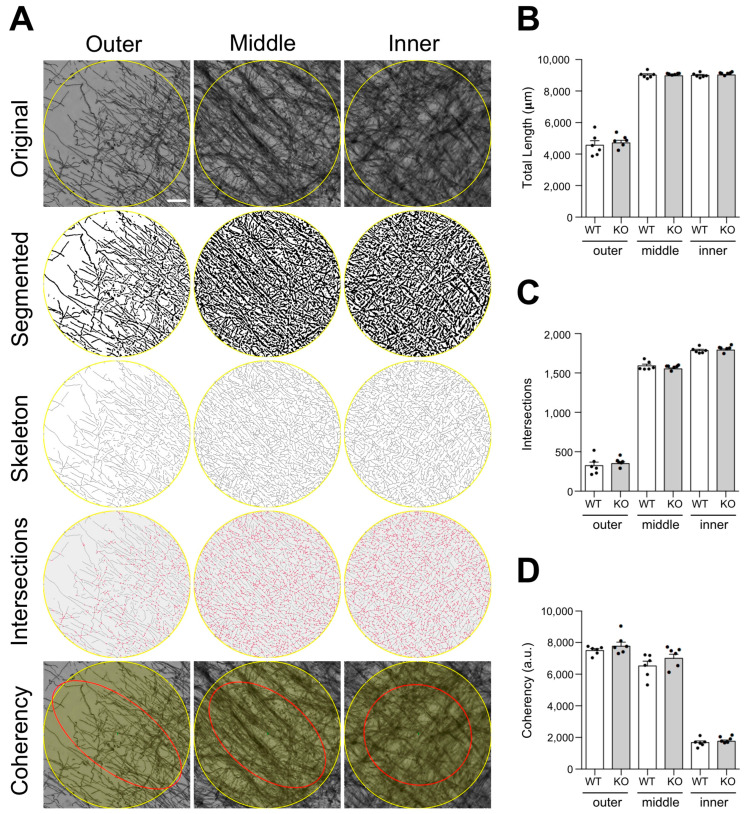
Myelin microstructural analysis of the cerebral cortex. (**A**) Top panels show representative microphotographs of the selected ROIs within each layer analyzed (Outer, Middle and Inner, Supplementary Figure S2). Segmented images, skeletonized masks and intersections measured are shown in descending order in the middle panels. Bottom panels show the best fitting ellipses (red), which are visual representations of the coherency (ellipse elongation) and orientation (ellipse direction) parameters. Coherency varies from maximum values in the outer layer where the ellipse becomes more elongated to minimum values in the inner layer where the ellipse becomes a circle. Bar graphs show the total myelin fiber length (**B**), number of intersections (**C**), and coherency values (**D**) measured for the outer, middle and inner layers of the cortex of WT and Lgals4-KO mice. Data are means + SEM (WT n = 6; KO n = 6). Dots in bar graphs represent the mean value of all the sections analyzed per animal. No significant differences between groups were observed for any parameter measured according to a Student’s *t*-test or Mann–Whitney test. (Scale bar = 20 μm).

To obtain a more detailed comparison of cortical myelin micro-distribution between WT and Lgals4-KO mice, an exhaustive study of MBP-stained tissue samples, combined with an in-depth image analysis was carried out (Figure 4). Several structural parameters of the myelinated axon were quantitated, such as the total myelinated fiber length (Figure 4B), the number of fiber intersections (Figure 4C) and the fiber coherency, which indicates to what extent myelinated fibers present an oriented distribution within the tissue (Figure 4D). Related to this latter parameter, we also compared the distribution curves of myelinated fiber orientations measured as positive/negative angles from the vertical (0 degrees) (Supplementary Figure S3). All parameters, measured in equivalent ROIs located in the inner, middle, and outer cortical segments (Figure 4A and Supplementary Figure S2), showed no significant differences between WT and Lgals4-KO mice in any of the cortical zones analyzed, indicating that the microstructure and complexity of cortical myelinated fibers are not altered in the absence of Gal-4.

In sum, data obtained from immunohistochemical experiments indicate that the lack of Gal-4 expression does not significantly alter somatosensory cortex myelination, according to unchanged expression, tissue distribution and microstructure of cortical myelin.

### 3.4. Unaltered Impulse Transmission along the Hippocampal Myelinated CA3-CA1 Projections in Lgals4-KO Mice

One of the main functions of myelin is to support the performance of the electric nerve impulse along the axons, regulating its conduction velocity. Therefore, we tested whether the absence of Gal-4 could affect myelin function at the conductivity level.

**Figure 5:**
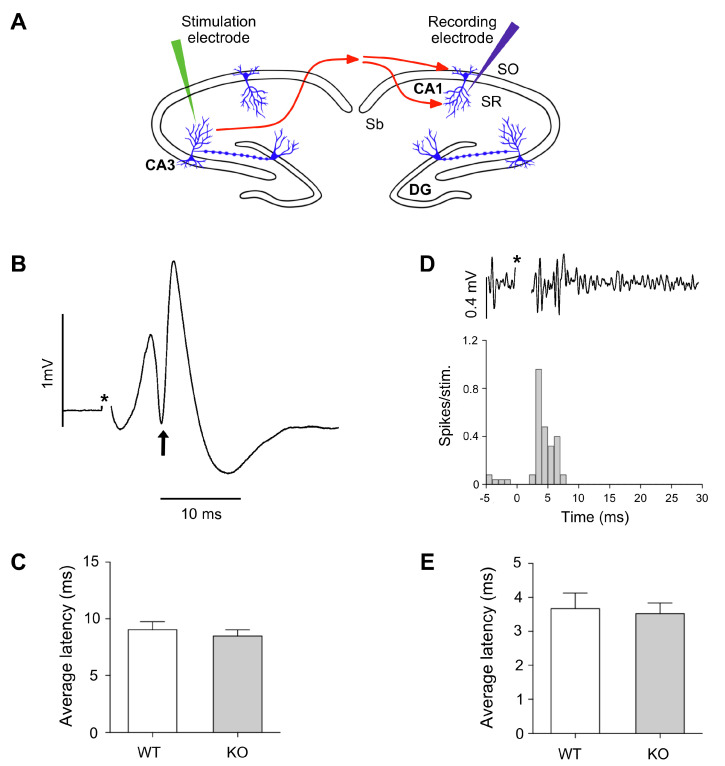
(**A**) Schematic representation of the in vivo electrophysiological setup. Stimulation electrodes were stereotactically located at CA3 hippocampal level of WT or Lgals4-KO mice, and recording electrodes were located at the CA1 region of the contralateral hemispheres. Latencies between stimuli and evocated neuronal responses were used to estimate the speed of impulse transmission along myelinated axon projections of the hippocampal commissure between these two regions. (**B**) Example of a local field potential recording of a populational spike in CA1 in response to CA3 stimulation (averaged from 32 stimuli). Latency was considered as the time between stimulation (*) and the negative peak of the populational spike (arrow). (**C**) Populational data from control group and Lgals4-KO group showing average latency values from peaks of populational spikes (error bars are SEM). (**D**) Example of multiunit activity showing action potentials in CA1 in response to stimulation in CA3 (upper trace). Peri-stimulus time histogram (PSTH) obtained from multiunit activity in CA1 in response to stimulation of CA3 (32 stim). Latency was considered as the first bin showing increased values above baseline (3 ms in the example). (**E**) Populational data from control group and Lgals4-KO group showing average latency values from first bin of response in PSTH (error bars are SEM). Note that in both recordings electrical artifact of the stimulation has been removed and marked by an asterisk (*).

Electrophysiological experiments were intended to measure the latencies of the CA1 response to the stimulation in contralateral CA3, in order to determine whether axonal conduction velocity of connecting myelinated fibers was different in Lgals4-KO animals with respect to WT mice (Figure 5A). The latency of the populational spike in CA1 is defined as the time between the stimulation and the maximal value of negative peak obtained from averaged responses to 30−50 stimuli (Figure 5B). According to this, the average latency of the populational spike obtained for the control group was 9.04 ms (SEM 0.70 ms) and for the Lgals4-KO group was 8.48 ms (SEM 0.55 ms). A Student’s *t*-test analysis of these results showed no significant differences in latencies measures to the negative peak of populational spike (*p* = 0.541) (Figure 5C).

A further approach was used to study possible alterations of conduction velocities along myelinated axons in Lgals4-KO animals. It is based on the study of the first action potentials latencies recorded from multiunit activity in CA1. As our recording electrode was placed in the pyramidal layer, action potentials were clearly detected (see methods), and we observed that a small population of neurons showed early (before the populational spike recorded with LFP) action potentials as responses to stimulation.

Therefore, we considered that these rapid and identifiable neuronal responses could precisely reveal alterations on conduction velocity of axons from CA3. When action potentials were extracted, a peri-stimulus-time-histogram (PSTH) was built to identify initial responses, as the first temporal point in which multiunit responses passed the threshold of background activity (Figure 5D). Based on this, the average latency of PSTH obtained in CA1 pyramidal layer in response to CA3 stimulation was 3.6 ms (SEM 0.47 ms) for control group, and 3.5 ms (SEM 0.31 ms) for the Lgals4-KO group. A Student’s *t*-test analysis showed that first responses based on action potentials were not altered in Lgals4-KO animals (*p* = 0.794) (Figure 5E).

### 3.5. Normal Motor Performance in Lgals4-KO Mice

Finally, we set out to evaluate the motor performance. To test this, mice from both strains were subjected to the open field behavioral test in order to evaluate basic motor performance.

**Figure 6:**
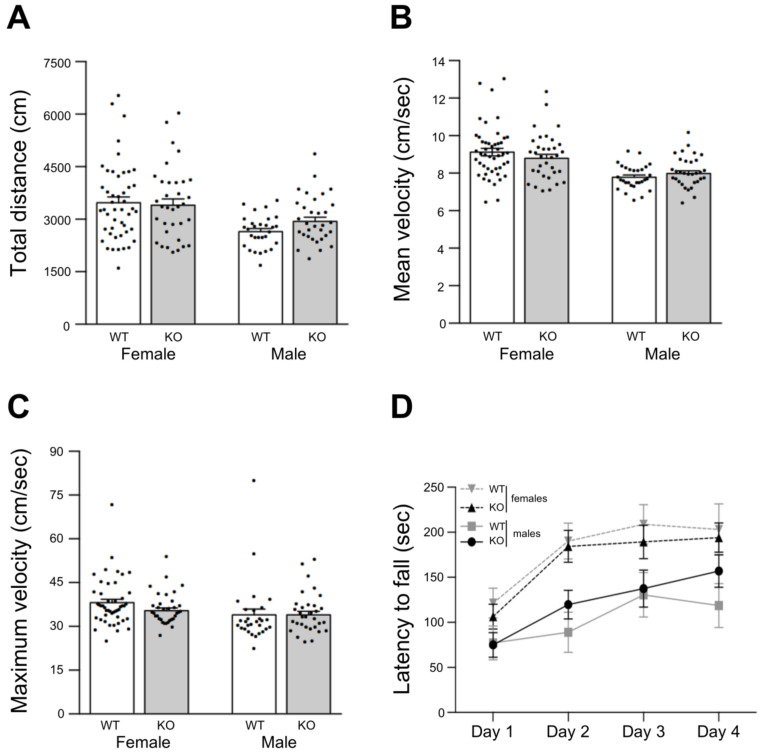
Spontaneous locomotion evaluated by the open field test. Total distance travelled (**A**), mean velocity (**B**) and maximum velocity (**C**) are displayed in bar graphs. Data are means + SEM (KO: n = 32 males, n = 34 females; WT: n = 29 males, n = 47 females). No differences between groups were observed according to two-way ANOVA followed by Tukey post hoc test. (**D**) Motor skill learning and motor performance evaluated by the rotarod test. The graph displays the time each animal takes to fall (latency) from an accelerating rotarod at different days after previous training. Data are mean latencies of three trials per day ± SEM. (KO: n = 5 males, n = 6 females; WT: n = 9 males, n = 12 females). Dots in bar graphs represent the measured value for each animal. No significant differences were detected between gender groups or mice strain groups according to two-way ANOVA with repeated measures followed by Bonferroni post hoc test.

Under such an experimental paradigm, total travelled distance (Figure 6A), and mean (Figure 6B) and maximum locomotion velocities (Figure 6C) tend to be higher for female mice when compared to males, but they did not show significant differences between mice strains.

In addition, motor skill learning and forced motor performance were assessed by the accelerating rotarod test. In this test, after a training period with static and slow constant rotation speed, the time to fall (latency) is recorded under increasing rotation speed (3 trials in 4 consecutive days). Typically, latencies rise until they reach a plateau after two days (learning period), and during the last two days of tests motor skills are assessed. In our case, male mice tend to show an extended learning period reaching the third day with low latency values (below 150 s) at the plateau (Figure 6D solid lines). In contrast, female mice present the typical 2 days learning period with higher latency values (200 s) at the plateau (Figure 6D dashed lines). In spite of this gender-related trend, there were no significant differences between gender groups or mice strain groups according to ANOVA and multiple comparisons analyses (Figure 6D).

These results showed that Lgals4-KO mice display a normal locomotion activity indicative of normal myelination, in good accordance with the observed electrophysiological features and biochemical/cellular studies.

Altogether, the presented results corroborate that myelination is not essentially compromised, either structurally or functionally, in a background of Gal-4 null expression. Nevertheless, the formation and regulation of differentiated axon membrane zones (NMS) is still relevant for myelin organization and function, as their occurrence and regulation are preserved even in the absence of some of their components. NMS in cultured neurons could constitute a trustable in vitro visualization of such axon membrane specializations.

## 4. Discussion

Different in vitro studies pointed to the participation of Gal-4 in myelination. Gal-4 has been proposed as an inhibitor of OLG maturation [20], or as a local impairment for myelin deposition in certain axon segments [21]. In contrast, Gal-4 was also reported to stimulate OLG myelin production [22]. Even though Gal-4 expression is very low within the nervous system, the reasoning that Gal-4 could either stimulate or inhibit myelination at different moments of neuron differentiation could not be ruled out. Nevertheless, to our knowledge no in vivo studies have approached the physiological relevance of Gal-4 in brain myelination so far. In this study we have used Gal-4-deficient mice to tackle this question. 

In addition to classical internode/node myelin structure, cortical neurons display diverse myelination patterns influenced by axon diameter and neuronal identity [4]. These patterns include long non-myelinated axon tracts proposed as high synaptic plasticity platforms in brain cortex [3,5,6]. Although the relation of NMS observed in cultured neurons [10,21,35] with those non-myelinated tracts has not been demonstrated, it is very likely that NMS reflect in non-permeabilized fixed axons the existence of portions of the axonal surface enriched in particular components that impair local myelin deposition. Besides the fact that NMS are not myelinated in co-cultures with OLGs [21], further indications that NMS can be considered a visualization of cortical myelin-free axon segments are the limited number of molecules described as NMS components (Figure 1 and Supplementary Table S1), and the detection of some molecules exclusively expressed at the complementary “non-NMS” axon segments, i.e., PSA-NCAM (Figure 1 and Supplementary Table S1). In this sense, binding of Maakia Amurensis lectin, with preferential specificity for sialylated glycans containing Neu5Ac/Gluα2-3-Gal sequence, also labels “non-NMS” axon segments (not shown). In addition, NMS are down-regulated as the differentiation of mice-derived cultured neurons proceeds in vitro, as it was previously shown for rat-derived neurons [21], indicating inter-species similarity in this aspect. In fact, to study eventual NMS formation and regulation in the absence of Gal-4, we have followed some of the NMS components identified in cultured rat cortical neurons (Figure 1A, and Supplementary Table S1), and observed that their formation and time-dependent regulation pattern occur similarly in cultured Lgals4-KO mouse cortical neurons and in WT neurons (Figure 1B,C), in contradiction to a possible involvement of Gal-4 in NMS regulation. In this sense, neuronal activity is another factor that can modulate myelination thus it could also play a role in NMS regulation [38]. Neuronal activity-dependent secretion has been proposed to instruct adjacent OLGs to form and stabilize the myelin sheath on selected axons, as shown by synaptic activity reduction using the voltage-gated Na+ channel blocker TTX [36,37]. Nevertheless, in those experiments all neurons were similarly affected by TTX, so the mechanism underlying the selection of a particular axon for myelination is not clear, although it has been reported that maintained suppression of neuronal activity selectively reduces the length of the internodes [39]. Importantly, we show here that TTX treatment of cortical neuron cultures induces the tendency to longer NMS than in untreated controls (Figure 1B,C, bar graph). This would leave shorter zones for myelin deposition, which could even mark this axon as a “bad choice” to be myelinated. In contrast, as discussed above, NMS length is reduced with neuron differentiation facilitating myelination whenever the axon is sufficiently developed. Given that our cultures are essentially devoid of OLGs, presented data suggest that the selection of “myelinable” axons or axon segments could be, at least in part, intrinsic to neuron differentiation and activity through NMS regulation, independent of a possible neuronal-driven stimulation of OLG differentiation towards their myelinating state. The fact that OLGs can myelinate fixed axons or synthetic fibers [41,42] in the absence of living neurons would support this idea. As in the case of NMS regulation by time in culture, our finding that NMS formation and regulation by neuronal activity is not affected in the absence of Gal-4 indicates, contrary to our initial hypothesis, that this lectin is not essentially involved in NMS regulation. Nevertheless, conserved NMS formation and regulation, even in the absence of Gal-4, maintain the possibility for the participation of these axonal structures in myelin organization.

NMS did not show any difference in vitro between WT and Lgals4-KO neurons; however, cortical myelin could still be altered in composition or in tissue distribution. Our aim was to evaluate whether constitutive Gal-4 absence (as is the case in Gal-4-KO mice) resulted in structural or functional abnormalities in mature myelin in vivo. Taking this into account, we chose P30 as the closest time point to perinatal period in which we could study fully structured and functional myelin. In contrast to the expected, no differences in the expression of main oligodendrocyte and myelin markers were observed between WT and Lgals4-KO mice cortical tissues, either at the transcriptional (Figure 2A) or at the protein (Figure 2B) levels. In order to accurately study the quantitative expression and distribution of main myelin markers in cortical tissue by immunohistochemistry means, we took into account the significant differences in myelin distribution between less and more densely myelinated regions [5] and subdivided the full cortex into three layers (Outer, Middle and Inner) of equal thickness (Supplementary Figure S2) that were independently analyzed and compared between mice strains. Consistently, oligodendrocyte number and quantitative distribution in cortical layers were similar (Figure 3A), as was the case for the quantitative expression and distribution of main myelin markers MBP and PLP (Figure 3B,C). 

Taking a step beyond, we performed an in-depth microstructural study analysis of MBP-stained tissue samples to assess any possible differences in cortical myelin complexity (Figure 4). First, structural parameters related to the complexity of the myelin microstructure such as the total myelinated fiber length (Figure 4B), and the number of fiber intersections (Figure 4C) did not present any inter-strain differences in any of the three cortical subzones. Importantly, these parameters do not account for the orientation of the myelinated fibers so differently oriented patterns were still possible. In order to check this, we calculated the fiber coherency (C), that indicates whether the image features are oriented or not. In brief, C equals 1 when the structure in the image shows a dominant orientation, and C equals 0 when the image is mainly isotropic, thus one can consider the coherency of myelinated fibers as an inverse measure of myelin microstructure complexity. In fact, C values are low in the inner cortical zone (highly complex) while they are high in less complex middle and outer zones, although no differences were observed between the two mice strains (Figure 4D). The same lack of significant differences was observed by expressing fiber orientation as a normalized distribution of angles (Supplementary Figure S3), which lead us to conclude that the microstructure and complexity of cortical myelinated fibers are normally established in brain of Gal-4-deficient animals.

NMS analysis in vitro, the expression of myelin markers and their histochemical distribution, together with the detailed orientation analysis of cortical myelinated segments, pointed to the conclusion that myelination is not altered in a Gal-4-deficient background compared to WT animals. Nevertheless, this did not preclude the possibility of functional myelin abnormalities that could be reflected by changes in the conduction velocity of the nerve impulse along the axons, or by related animal motor behavior alterations. In this sense, axonal conduction velocity along the myelinated interhemispheric hippocampal commissure, measured as the latencies of the CA1 response to the stimulation in contralateral CA3 (Figure 5B,C), or as the latencies of the early action potentials recorded from multiunit activity in CA1 (peri-stimulus response, Figure 5D,E), showed no differences between both strains. Consistently, basic motor parameters such as travelled distance and mean or maximum velocities measured in the open field (Figure 6A–C), as well as latency to fall measured at the rotarod (Figure 6D), presented no significant differences when comparing WT to Lgals4-KO animals.

In sum, and in spite of previous in vitro evidence that strongly suggested a central involvement of Gal-4 in myelination, all the results presented in this work demonstrate that in vivo cortical myelin of Gal-4-deficient mice does not present any differences in composition, distribution, organization or function when compared to control wild-type animals. Anyway, the fact that we did not detect differences in mature myelin of KO mice does not preclude the possibility of differences during the process of myelination (maybe an accelerated myelin deposition in Gal-4-KO mice?) leading to the same final state. Such a situation has been reported for BTBR mice strain, an autism spectrum disorder mouse model, in which neonatal frontal brain myelination is increased, associated with elevated levels of MBP. In contrast, myelin pattern appears unaltered in adult BTBR mice, suggesting an accelerated developmental myelination [43].

Our results could be explained either by a negligible role of Gal-4 in myelination, compatible with the particularly low Gal-4 expression in the nervous system, or by the contribution in vivo of some mechanism/s to counteract the absence of Gal-4 during myelin maturation to render a normal mature myelin.

Among others, such a compensating mechanism could rely on the role of Gal-4-related galectins. While most mammalian species, including humans, have a single Gal-4 encoding gene (Lgals4), some mice strains have two paralogues: Lgals4 and Lgals6. The latter encodes for galectin-6 (Gal-6) that, as Gal-4, is a tandem-repeat type galectin and is also prominently expressed in the intestine, presenting both a remarkable functional redundancy [44,45], making Gal-6 a suitable candidate to compensate the lack of Gal-4. Nevertheless, we rapidly eliminated such possibility as the Lgals4-KO mice were obtained from the C57BL/6NJ strain that lacks the Lgals6 gene and therefore does not express Gal-6 protein [45]. 

Galectin-8 (Gal-8) is another tandem-repeat type galectin closely related to Gal-4 [45]. Both lectins have been proposed to provide a unique form of innate immunity against molecular mimicry by specifically targeting microbes bearing glycan self-like antigens in the digestive tract [18,46]. In spite of the fact that Gal-8 is expressed in mouse brain (Supplementary Figure S1B), no function for Gal-8 in the nervous system has been clearly established. Its expression, as well as that of Gal-4, is increased in multiple sclerosis brain tissue [40,47] and down-regulated in the hippocampus of schizophrenia patients [48], but studies involving Gal-8 KO mice have mainly focused the roles of Gal-8 in cancer or immunity [49,50] neglecting possible neurologic implications. Further studies are thus required to assess the eventual relevance of Gal-8 in myelin regulation and whether it could compensate for the absence of Gal-4 in myelination.

## Data Availability

The data generated are available upon reasonable request to the corresponding authors.

## Supplementary Materials

The following supporting information can be downloaded at: https://www.mdpi.com/article/10.3390/cells11213485/s1, Figure S1: Lgals4-KO mice characterization; Figure S2: Somatosensory cortex segmentation for myelin analysis; Figure S3: Mean normalized distribution of myelinated fiber orientation; Table S1: Potential components of the NMS; Table S2: TaqMan probes used for RT-PCR; Table S3: Detailed information about antibodies used in this work.
