# Supplementary material for: Normal Cortical Myelination in Galectin-4-Deficient Mice
Source: Cells. 2022 Nov 3;11(21):3485. doi: 10.3390/cells11213485 (PMC9658391; doi:10.3390/cells11213485)
Supplement: Supplementary file 1 [file cells-11-03485-s001.zip › cells-1939428-supplementary.pdf]

Supplementary Material to:

Normal Cortical Myelination in Galectin-4-Deficient Mice

María-Elvira Brocca[1,†], Arancha Mora-Rubio[1,†], Elena Alonso-Calviño[2], Elena Fernández-López[2], Natalia Díez-Revuelta[1], David Martos-Puñal[1], Juan Aguilar[2], Alonso M Higuero[1,*] and José Abad-Rodríguez[1,*]

Supplementary Figure S1

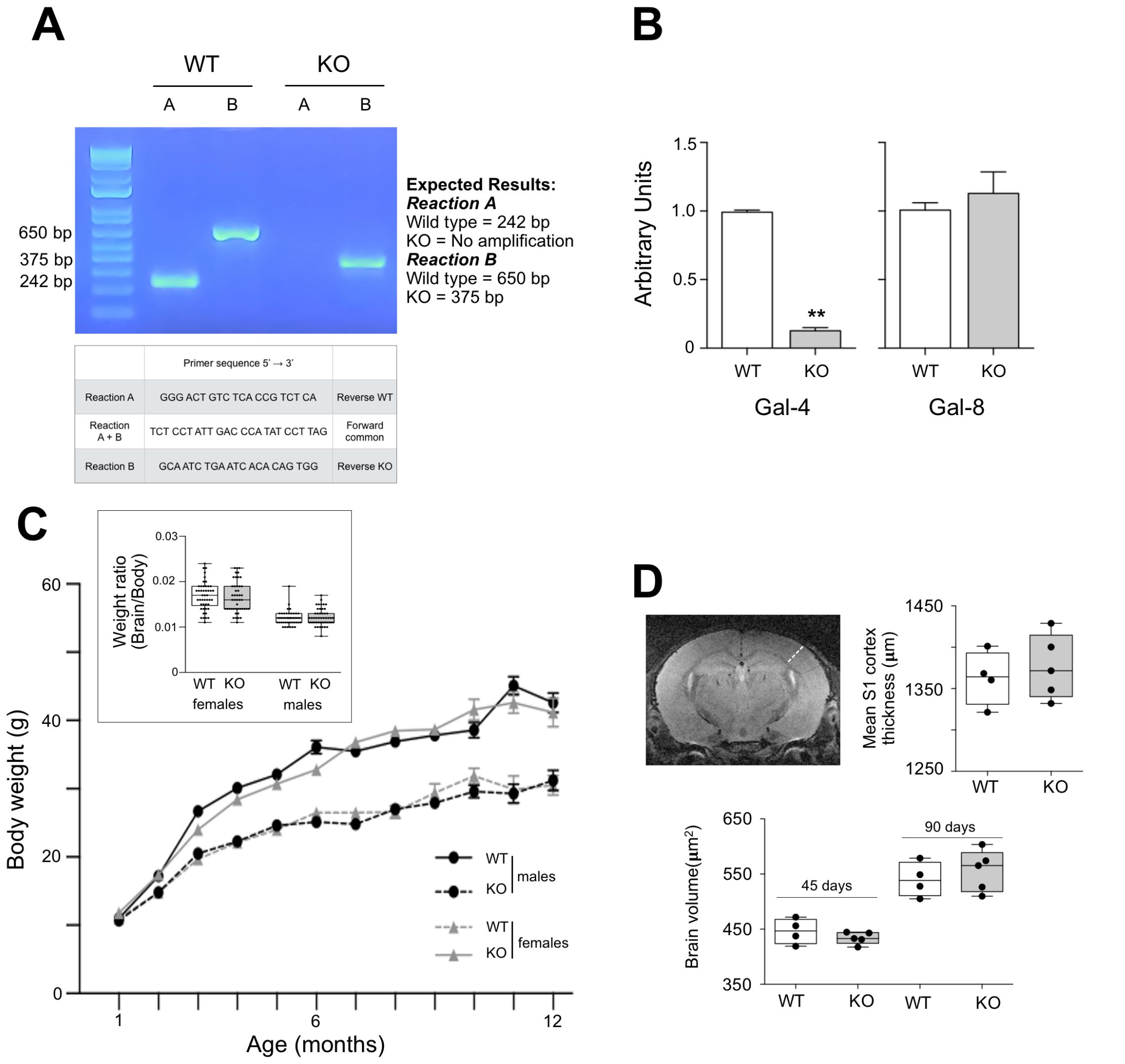

Supplementary Figure S1

Lgals4-KO mice characterization. (A) Representative genotyping of wild type C57BL/6NJ mice (WT) and C57BL/6NJ-Lgals4em1(IMPC)J/J mice (KO). (B) Expression of galectins 4 and 8 in brain cortex of both mice strains measured by RT-PCR (values are means + SEM, n=5, **p<0.01 Student's t-test). (C) Mice body weight evolution with age segregated by gender and strain. No significant differences were observed when comparing mice strains by two-way ANOVA analysis (values are means ± SEM; WT: females n=151, males n=158; KO: females n=169, males n=150). The inset displays the brain weight/body weight ratio of animals aged from 3 to 12 months. Data are arranged as a min. to max. box plot (WT and KO females n=46, WT males n=48; KO males n=49). There are no differences between mice strains. (D) Comparative analysis of WT (n=4) vs KO (n=5) mice S1 cortex thickness and brain volume measured by NMR imaging. Data are arranged as a min. to max. box plot. No significant differences between groups were observed by Student's t-test.

Supplementary Figure S2

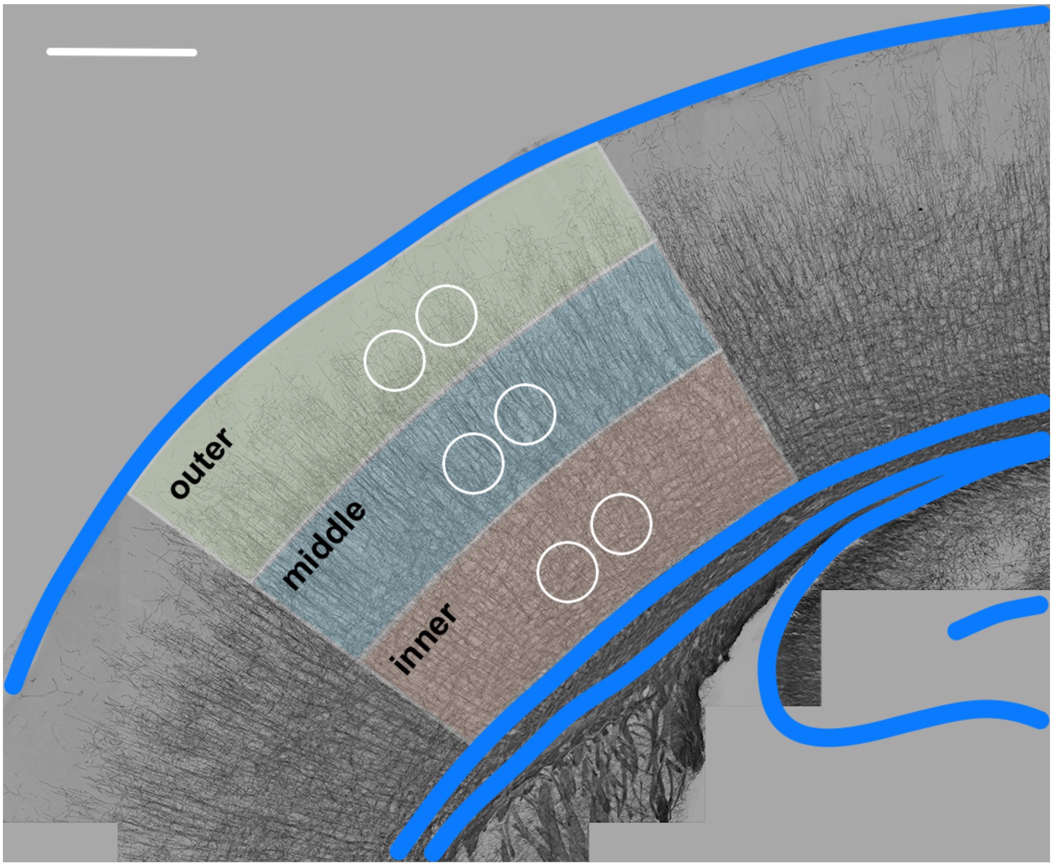

Supplementary Figure S2
Somatosensory cortex segmentation for myelin analysis. Cortex of MBP-immunostained mice brain slices subdivided into three layers (Outer, Middle and Inner) of equal thickness according to increasing complexity of myelin fiber distribution. A similar division was used with Olig2 and PLP1-immunostained tissue in order to quantify the segmented expression of myelin markers as shown in Fig. 3. White circles are representative examples of selected ROIs used for myelin complexity studies shown in Fig. 4. (Scale bar 500 μm).

Supplementary Figure S3

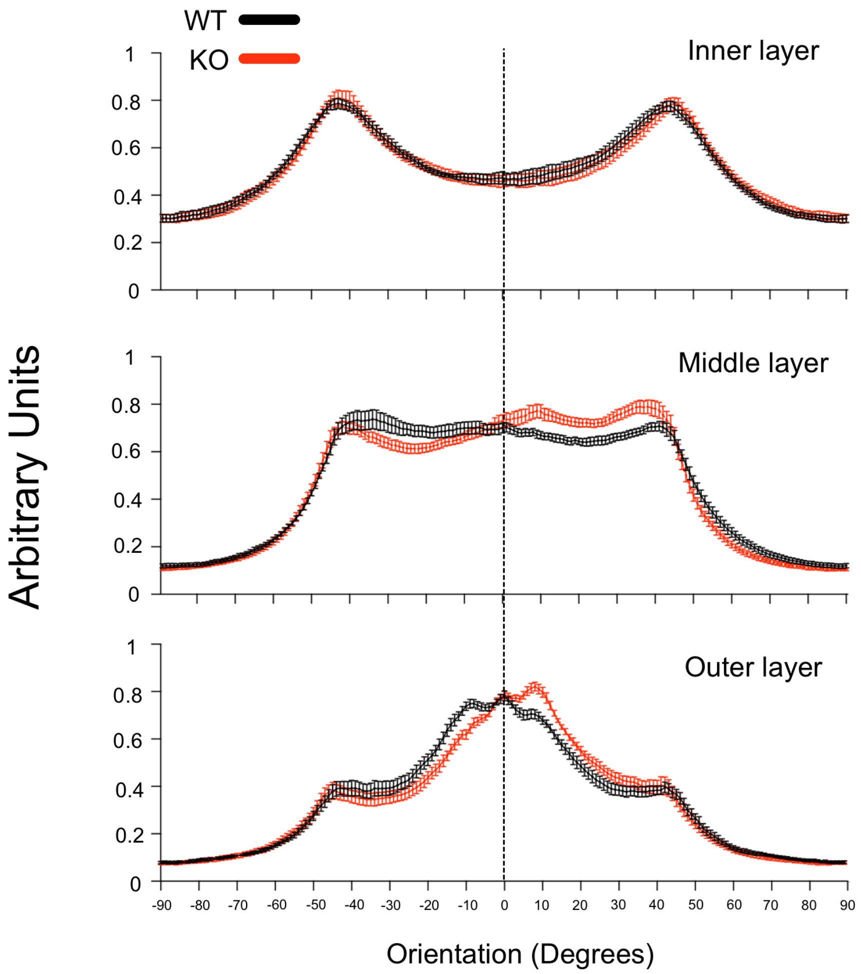

Supplementary Figure S3

Mean normalized distribution of myelinated fiber orientations for the inner layer (upper panel), middle layer (middle panel), and outer layer (bottom panel) of the somatosensory cortex of WT and Lgals4-KO mice. Normalized frequencies are shown at 1 degree intervals. Data are means + SEM (WT n=6; KO n=6). No significant differences between groups were observed in any of the three layers analyzed according to a two-way repeated measures ANOVA.

Method	Molecule	G4D localiz.
IP + proteomics	Myosin IIb	**n.d.**
	**Drebrin E**	**+**
	Tubulin β5	**-**
	Cytoplasmatic Actin 2	**-**
Co-immunofluorescence; Nodes/paranodes markers	**Caspr**	**+**
	**Contactin 1**	**+**
	Neurofascin 186	**-**
	Sodium channels (Pan-Nav)	**-**
Co-immunofluorescence; Anionic glycoconjugates	**Heparan sulfate Proteoglycan (mixed)**	**+**
	Chond. sulfate proteoglycan (mixed)	**-**
	Chond. sulfate A/C (4[6]- monosulfate)	**-**
	**Chond. sulfate D/E (4,6- disulfate]**	**+**
	Neuroglycan-C (CSPG5; mixed chond. sulfate)	**-**
	HNK1	**-**
	PSA-NCAM*	**-**
Raft proteomics	**VDAC1 (porin)**	**+**
	α-internexin	**-**
	Mitochondrial 60KDa HSP	**n.d.**
	Dihydropyrimidase-related prot. 2	**n.d.**

Supplementary Table S1. Potential components of the NMSs. Several methods were used to identify molecular candidates to be components of the NMSs: i) Crosslinking and immunoprecipitation (IP) and proteomic analysis. Membrane molecules from cultured cortical neurons were crosslinked with reversible crosslinkers that do not cross the plasma membrane. The postnuclear lysates obtained were immunoprecipitated with anti-Gal-4 antibodies and the immunoprecipitates were analyzed by LC-MS/MS (proteomics); ii) Co-localization by immunofluorescence of node/paranode components and anionic glycoconjugates with Gal-4 under non-permeabilizing conditions and, iii) Proteomic analysis of membrane raft fractions purified from neurons in culture by density gradients and chloroform/methanol precipitation. In the right column, those molecules whose expression co-localizes in the NMSs are indicated with "+" (Fig. 1A). (*PSA-NCAM is highlighted in blue to indicate that it is expressed in axonal segments alternative to NMSs; Fig. 1A)

Gen	Reference
Lgals4 G07	Mm01179060_m1
Lgals8	Mm01332239_m1
SOX10	Mm00569909_m1
MAL	Mm01339780_m1
MBP	Mm01266402_m1
Olig2	Mm01210556_m1
PLP1	Mm01297210_m1
GAPDH	Mm99999915_g1
β-Actin	Mm02619580_g1

Supplementary Table S2. TaqMan probes (Applied Biosystems) used in expression measurements by RT-PCR. All probes were conjugated to fluorescein amidite (FAM) fluorophore .

Antigen	Host	Clone	Cat. No.	Source	IHQ/IF/IC working dilution	WB working dilution
Beta-Actin	M	AC-15	sc-69879	Santa Cruz		1/10000
Caspr	G	Polyclonal	sc-11174	Santa Cruz	1/200	
CS-D/E	M	MO-225	AMS.A2872	AMSBIO	1/100	
CS-A/C	M	CS-56	C8035	Sigma	1/100	
Drebrin E	R	EPR12634	Ab178408	Abcam	1/200	
Galectin-4	M	B-9	sc-271533	Santa Cruz	1/200	
Galectin-4	M	E-2	sc-271209	Santa Cruz	1/200	
Galectin-4	R	Polyclonal		provided by Gabius H.J.	1/200	
GAPDH	M	6C5	MAB374	Merck Millipore		1/10000
HNK1	M	TB01	M7271	Dako	1/200	
HS	M	F58-10E4	370255	AMSBIO	1/100	
MAG	G	Polyclonal	Sc-9544	Santa Cruz		1/1000
MBP	Rt	12	Ab7349-1	Abcam	1/200	1/1000
MOG	R	E5K6T	96457			1/1000
Na^+ channel (PAN)	M	K58/35	S8809	Sigma	1/200	
Neuroglycan-C	G	Polyclonal	AF5665	RD Systems	1/200	
Neurofascin-186	M	A12/18	75-172	NeuroMab	1/200	
Olig2	M	211F1.1	MABN50	Merck Millipore	1/500	1/2000
PLP1	R	E9V1	28702S	Cell Signaling	1/200	
PSA-Ncam	M	2-2B	MAB5324	Merck Millipore	1/200	
βIII-Tub (Tuj1)	M	2G10	T8578	Sigma	1/1000	1/5000
VDAC1	R	D73D12	4661	Cell Signaling	1/200	
M-IgG Alexa Fluor 488	D		A21202	Invitrogen	1/500	
R-IgG Alexa Fluor 488	D		A21206	Invitrogen	1/500	
M-IgG Alexa Fluor 594	D		A21203	Invitrogen	1/500	
G-IgG Alexa Fluor 488	D		A11055	Invitrogen	1/500	

R-IgG Alexa Fluor 594	D	A21207	Invitrogen	1/500	
R-IgG biotinylated	G	BA-1000	Vector	1/500	
Rat-IgG biotinylated	G	629540	Zymed	1/500	
M-IgG HRP	S	NA931V	GE Healthcare		1/5000
R-IgG HRP	D	NA-934V	GE Healthcare		1/5000
G-IgG HRP	R	A8919	Sigma		1/5000
Rat-IgG HRP	R	A9542	Sigma		1/5000

Supplementary Table S3. Detailed information about primary and secondary antibodies used for immunohistochemistry (IHQ), immunohistofluorescence (IF), immunocitochemistry (IC) and western blot (WB). M, mouse; R, rabbit; Rt, rat; D, donkey; H, horse; S, sheep; G, goat.
